# An 8-gene signature for prediction of prognosis and chemoresponse in non-small cell lung cancer

**DOI:** 10.18632/oncotarget.13357

**Published:** 2016-11-15

**Authors:** Muhammad Shahid, Tae Gyu Choi, Minh Nam Nguyen, Abel Matondo, Yong Hwa Jo, Ji Youn Yoo, Ngoc Ngo Yen Nguyen, Hyeong Rok Yun, Jieun Kim, Salima Akter, Insug Kang, Joohun Ha, Chi Hoon Maeng, Si-Young Kim, Ju-seog Lee, Jayoung Kim, Sung Soo Kim

**Affiliations:** ^1^ Department of Biomedical Science, Graduate School, Kyung Hee University, Seoul, Republic of Korea; ^2^ Department of Biochemistry and Molecular Biology, Medical Research Center for Bioreaction to Reactive Oxygen Species and Biomedical Science Institute, School of Medicine, Kyung Hee University, Seoul, Republic of Korea; ^3^ Department of Medical Oncology and Hematology, Kyung Hee University Hospital, Seoul, Republic of Korea; ^4^ Department of Systems Biology, Division of Cancer Medicine, The University of Texas MD Anderson Cancer Center, Houston, Texas, USA; ^5^ Departments of Surgery and Biomedical Sciences, Cedars-Sinai Medical Center, Los Angeles, CA, USA

**Keywords:** non-small cell lung cancer, microarray analysis, prognosis, chemosensitivity

## Abstract

Identification of a potential gene signature for improved diagnosis in non-small cell lung cancer (NSCLC) patient is necessary. Here, we aim to establish and validate the prognostic efficacy of a gene set that can predict prognosis and benefits of adjuvant chemotherapy (ACT) in NSCLC patients from various ethnicities. An 8-gene signature was calculated from the gene expression of 181 patients using univariate Cox proportional hazard regression analysis. The prognostic value of the signature was robustly validated in 1,477 patients from five microarray independent data sets and one RNA-seq data set. The 8-gene signature was identified as an independent predictor of patient survival in the presence of clinical parameters in univariate and multivariate analyses [hazard ratio (HR): 2.84, 95% confidence interval CI (1.74-4.65), *p=*3.06e-05, [HR] 2.62, 95% CI (1.51-4.53), *p=*0.001], respectively. Subset analysis demonstrated that the 8-gene signature could identify high-risk patients in stage II-III with improved survival from ACT [(HR) 1.47, 95% CI (1.01-2.14), *p=*0.044]. The 8-gene signature also stratified risk groups in *EGFR*-mutated and wild-type patients. In conclusion, the 8-gene signature is a strong and independent predictor that can significantly stratify patients into low- and high-risk groups. Our gene signature also has the potential to predict patients in stage II-III that are likely to benefit from ACT.

## INTRODUCTION

Lung cancer (LC) is one of the leading causes of cancer-associated deaths worldwide [1]. LC is broadly divided into two main groups: small cell lung cancer (SCLC) and non-small cell lung cancer (NSCLC). NSCLC accounts for 85% of all lung cancer cases, for which improvement of 15.9% has been reported in 5-year survival rate during the past few decades [2]. NSCLC is currently subdivided into two predominant histologic phenotypes: adenocarcinoma (ADC; 50%) and squamous cell carcinoma (SQC; 40%) [3, 4].

The current American Joint Committee on Cancer (AJCC) staging system serves as the best predictor of prognosis and a standard to guide treatment decisions in NSCLC [5]. Complete surgical resection is the most effective for patients in the early stage [6], even though 30-60% of patients diagnosed with stage IB to IIIA relapse and die within 5-year of survival [7]. For patients in stage II-III, adjuvant chemotherapy (ACT) is the standard treatment with survival rate from 4% to 15% [8, 9]. However, due to the heterogeneous nature of NSCLC, the current AJCC staging cannot accurately classify patients who would benefit from chemotherapy [10]. Prognostic biomarkers with transcriptomic data and the mutation status of genes which are important in cancer development need to be investigated [11]. Previous studies identified three major genes (*EGFR*, *KRAS*, and *ALK*) for the development of lung cancer [12–15]. Mutations in the *EGFR* have been associated with enhanced overall survival, whereas *KRAS* mutations may predict shorter survival for lung adenocarcinoma patients [16]. Molecular tests for these prognostic biomarkers have been started for preclinical and clinical applications to advance the treatment of NSCLC [17–20].

Recent advances in microarray gene expression profiling have demonstrated possibility of screening gene expression signatures to predict the prognosis of patients. Previously, this approach successfully identified prognostic and predictive gene signatures in the breast cancer [21]. To date, several studies based on gene expression signatures have been shown to classify various cancer patients into different prognostic groups with distinct clinical features by supervised or unsupervised methods [22–28]. However, the identified survival-related signatures lack consistency among studies, likely due to genetic alteration among patients, technical factors such as differences in microarray platforms, and limited number of patients. Therefore, it is important to establish a prognostic gene signature that could predict patient's survival and guide decisions of adjuvant therapy for individual patient.

In this study, we identified an 8-gene signature to distinguish two prognostic groups (low- vs high- risk), using an unbiased gene expression profiling and bioinformatics analysis. The 8-gene signature was further validated in five microarray retrospective and independent data sets and one RNA-seq data set. Furthermore, we assessed the associations of the identified prognostic gene signature with clinicopathological factors and molecular alterations. Finally, we investigated whether our 8-gene signature could predict patients who might have benefits from ACT in the patients diagnosed as stage II-III NSCLC. Our findings suggest that the 8-gene signature can be rapidly implemented in a clinical setting and demonstrated excellent predictive power for NSCLC.

## RESULTS

### Development of a prognostic gene signature and a risk predictor

**Figure 1:**
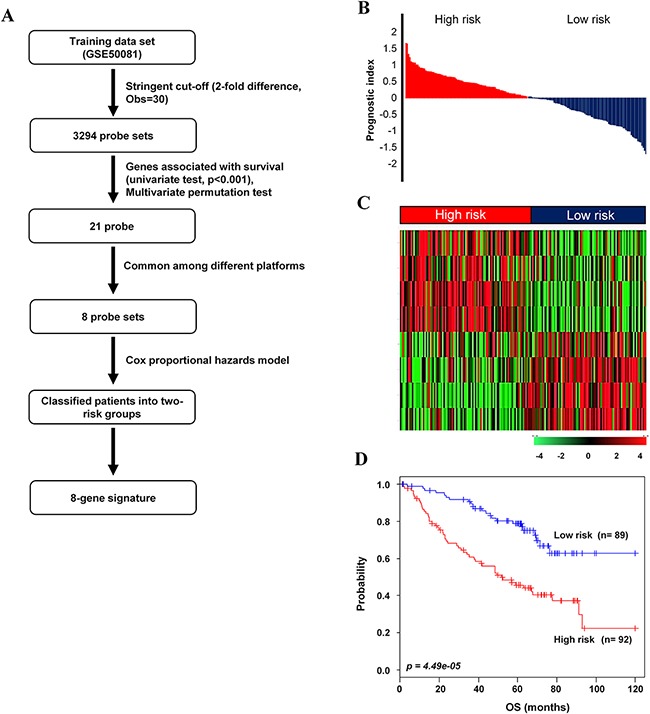
Survival analysis of the training data set **A**. Schematic overview of the procedure used to construct the 8-gene signature based on gene expression data. **B**. The relative prognostic index based on the 8-gene signature expression of each patient. **C**. The heatmap of the median centered 8 genes’ expression profiles (red, relative high expression; green, relative low expression) between low- and high-risk groups. **D**. Kaplan-Meier plots for OS of two risk groups in the training data set. The *p* values were computed by log-rank test.

In order to identify a prognostic gene signature that distinguished low- and high-risk NSCLC patients, gene expression profiling was analyzed in relation to survival data. GSE50081 was used as the training data set. As shown in the flow chart of the procedure (Figure 1A), after filtering for probe set intensity, 3,294 probe sets were analyzed in univariate Cox regression analysis with overall survival (OS) as the prognostic survival end point. A gene signature with 21-probe set was developed. However, the microarray chip type for the GSE50081 [29], GSE31210 [25, 30], GSE30219 [31], GSE29013 [32] and E-MTAB-923 data sets was Affymetrix GeneChip Human Genome U133 Plus 2.0 (HG-U133_Plus_2), and the other chip types for the GSE68465 and GSE42127 were Affymetrix GeneChip Human Genome (HG-U133A) and Illumina HumanWG-6 v3.0 Expression BeadChip (IlluminaHuman-WG6 V3), respectively, *as described in ‘Methods’ section (Supplementary Table S1)*. Among the 21-probe set, 8 probes, which were corresponded to 8 annotated genes, were commonly found both in the training and all validation data sets. Thus, this model was termed the 8-gene signature, including *STAT1, CLU, GTSE1, NUSAP1, ABCA8, TNNT1, ENTPD3* and *CPA3* (Supplementary Table S2). Prognostic index for each patient was calculated based on the 8-gene signature (Figure 1B). Patients were dichotomized according to the risk score into low (n=89) and high (n=92) risk groups on their prognostic index in the training data set. The heatmap showed different expression patterns of the 8-gene signature for the low- and high-risk patient groups into two clusters (Figure 1C). The Kaplan-Meier analysis confirmed that overall survival rate was different between the predicted low- and high-risk groups based on the 8-gene signature (*p=*4.49e-05, Figure 1D).

### The 8-gene signature can be used as an independent clinical parameters

We next tested whether the prognostic gene signature was associated with clinical parameters, including age, gender, smoking, stage and survival. Chi-square (χ2) test revealed that patient survival time (*p=*4.02e-5), stage (*p=*0.006) and smoking (*p=*0.003) were significantly correlated with our signature, while other parameters were not associated (Supplementary Table S4). To evaluate the prognostic accuracy of the 8-gene signature on overall survival (OS), univariate and multivariate Cox proportional regression analyses were performed in the training data set. In univariate and multivariate analyses, the stage was significantly associated with OS (HR: 1.68, 95% CI 1.04-2.71, *p=*0.031 and HR: 1.81, 95% CI 1.08-3.01, *p=*0.023, respectively). Univariate and multivariate analyses also showed that the 8-gene signature had the stronger prognostic ability than stage (HR: 2.84, 95% CI 1.74-4.65, *p=*3.06e-05 and HR: 2.62, 95% CI 1.51-4.53, *p=*0.001, respectively) (Table 1). No significant difference was obtained in other parameters.

**Table 1:** Univariate and multivariate Cox proportional hazard regression analyses in the training set

Variable	Univariate			Multivariate		
	HR	95% CL	*p*-value	HR	95% CL	*p*-value
**Age**	1.25	0.60-2.61	0.547	1.02	0.48-2.18	0.941
**Gender**	0.51	0.31-0.84	0.008	0.58	0.33-0.99	0.047
**Smoking (N vs E)^a^**	1.38	0.65-2.91	0.389	0.79	0.36-1.71	0.552
**Stage (I, II)**	1.68	1.04-2.71	0.031	1.81	1.08-3.01	0.023
**8-gene signature**	2.84	1.74-4.65	3.06e-05	2.62	1.51-4.53	0.001

^a^N; Never smoking, E; Ever smoking

### The 8-gene signature was validated in five independent data sets

**Figure 2:**
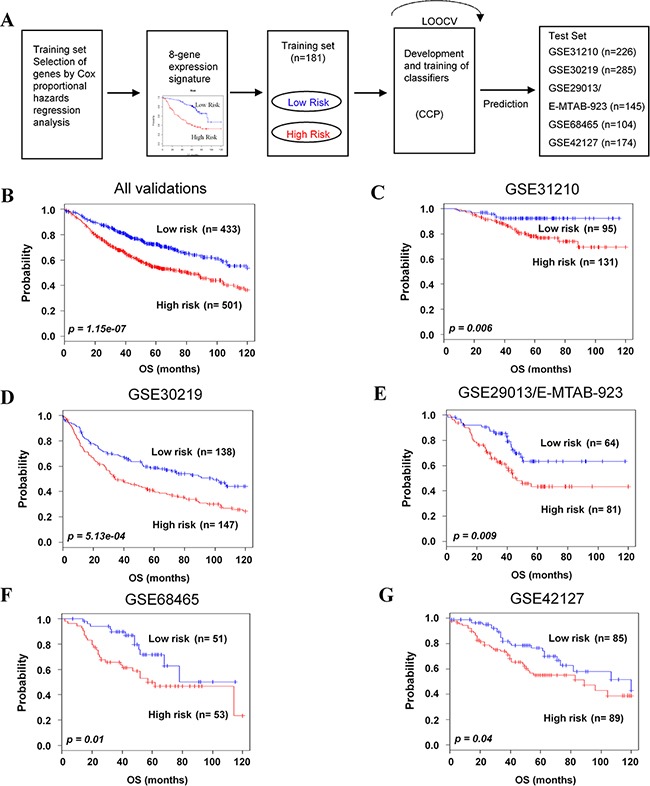
Validation of the 8-gene signature classifying independent data sets **A**. Schematic overview of the strategy used for the construction of the prediction model and evaluation of predicted outcomes in five independent data sets by the 8-gene signature. **B**. All combined validation data sets. **C-G**. GSE31210, GSE30219, GSE29013/E-MTAB-923, GSE68465, and GSE42127 were classified by the 8-gene signature into low- and high-risk groups, and evaluated by Kaplan-Meier analyses. The *p* values were computed by log-rank test.

To evaluate the robustness of the newly identified 8-gene classifier, validation was done on five independent microarray and one RNA-seq data sets of NSCLC. A flow chart of the procedure used to validate the external data sets is summarized in Figure 2A. The leave-one-out cross-validation (LOOCV) in five validation data sets resulted in the specificity and the sensitivity of 0.972 and 0.932, respectively. To identify whether the gene signature could be a more accurate prediction marker, we validated in the combined five validation data sets. As expected, the 8-gene signature significantly stratified patients into low- and high-risk groups (*p=*1.15e-07, Figure 2B). The three validation data sets (GSE31210, GSE30219 and GSE29013/E-MTAB-923) were derived from the same platform as the training data set. The 8-gene signature significantly classified patients into low- and high-risk groups for these data sets (*p=*0.006, *p=*5.13e-04 and *p=*0.009, Figure 2C-2E), respectively. Furthermore, cross-platform validation of the gene signature was demonstrated in two data sets. The Kaplan-Meier plots also predicted significant differences in prognosis among independent validation data sets: GSE68465 (*p=*0.01, Figure 2F) and GSE42127 (*p=*0.04, Figure 2G). Low- and high-risk groups were distinguished, based on the prognostic index of each patient (Supplementary Figure S1A-S1E). We also validated RNA-seq data from TCGA based on the 8-gene signature (*p=*0.005, Supplementary Figure S2). Moreover, univariate and multivariate analyses demonstrated that the 8-gene signature was a prognostic factor in combined validation sets (HR: 1.77, 95% Cl 1.43-2.20, *p=*1.71e-7 and HR: 1.34, 95% Cl 1.02-1.77, *p=*0.034, respectively) (Supplementary Table S5).

### Prognostic value of the 8-gene signature in association with stages

**Figure 3:**
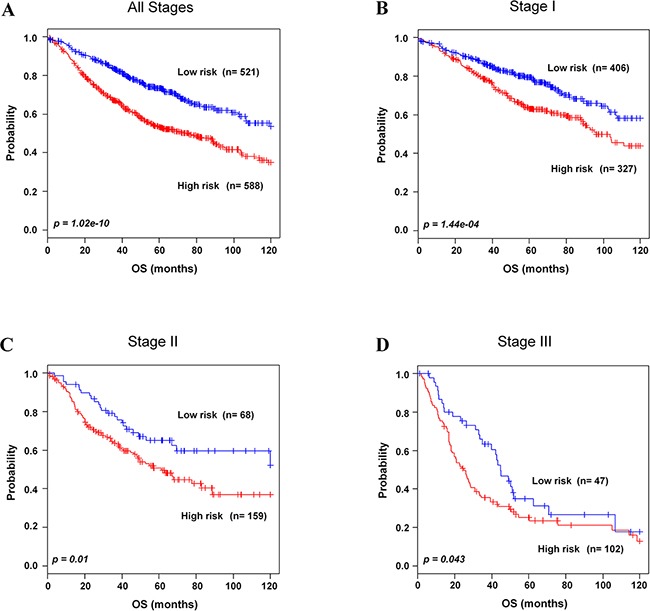
Kaplan-Meier survival analysis of the 8-gene signature with stages **A**. Patients in all stages in the combined training and validation data sets. **B**. Patients in stage I in the combined training and validation data sets. **C**. Patients in stage II in the combined training and validation data sets. **D**. Patients in stage III in the validation data sets were classified by the 8-gene signature into low- and high-risk groups. The *p* values were computed by log-rank test.

To evaluate whether the 8-gene signature could classify patients in each stages into two risk groups in the training and validation data sets, patients in each stage were combined as followed: stage I (n=733), II (n=227), and III (n=149). As expected, the 8-gene signature clearly stratified patients into low- and high-risk groups in combined patients in stage I-III (*p=*1.02e-10, Figure 3A; *p*=1.43e-11 in 5-year OS, Supplementary Figure S4A). Moreover, the 8-gene signature significantly separated stage I NSCLC patients into low- (n=406, 55.3%) and high-risk groups (n=327, 44.6%) (*p=*1.44e-04, Figure 3B; *p*=1.65e-05 in 5-year OS, Supplementary Figure S4B). In addition, our gene signature classified patients in stage II and III into low- and high-risk groups (*p=*0.01 and *p=*0.04, Figure 3C and 3D; *p*=0.0371 and *p*=0.0268 in 5-year OS, Supplementary Figure S4A and S4D, respectively).

### The 8-gene signature predicts clinical outcomes for adjuvant chemotherapy

**Figure 4:**
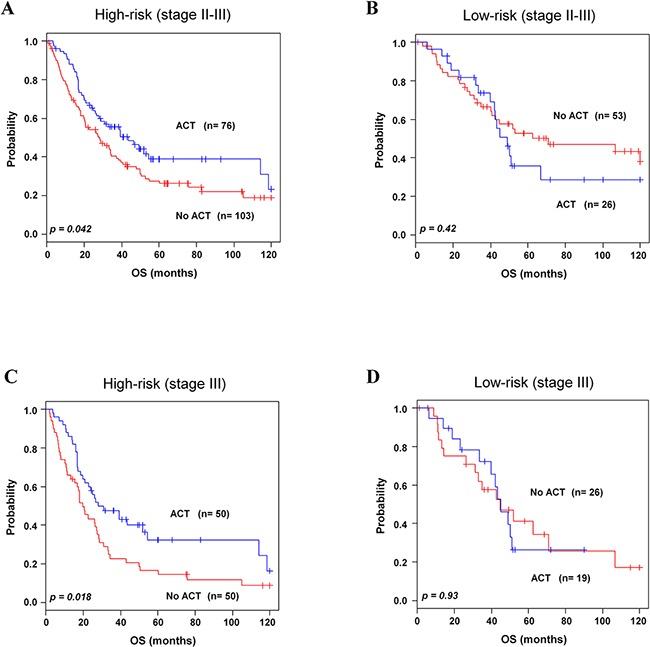
Kaplan-Meier survival analysis of the 8-gene signature with adjuvant chemotherapy Patients from combined validation data sets with available ACT data were included for analysis. **A-B**. Patients in high-and low-risk groups with chemotherapy in stage II-III. **C-D**. Patients in high- and low-risk groups with chemotherapy in stage III. Patients were plotted according to presence and absence of ACT. The *p* values were computed by log-rank test.

For NSCLC patients of stage II-III, ACT has improved survival rate and has become standard therapy [8, 9]. To find association of the 8-gene signature with response to chemotherapy, subset analysis was performed in stage II-III patients. By incorporating the 8-gene signature into chemotherapy information, the combined patients in stage II-III with high-risk group showed better survival with chemotherapy compared to without chemotherapy. In high-risk group, seventy six (42.4 %) patients improved survival from chemotherapy (*p=*0.04, Figure 4A; *p*=0.0382 in 5-year OS, Supplementary Figure S5A). On the contrary, low-risk group patients in stage II-III did not get any significant benefit from chemotherapy (*p=*0.42, Figure 4B. Similarly, among high-risk group of stage III, fifty (50 %) patients had benefit from chemotherapy (*p=*0.01, Figure 4C; *p*=0.0218 in 5-year OS, Supplementary Figure 5SB), which was not observed among low-risk group patients of stage III (*p=*0.93, Figure 4D). Our gene signature was also applied to patients with stage I or stage II. We found that all patients in these stages did not get benefit from chemotherapy (Supplementary Figure S3A-S3D).

### Association of the 8-gene signature with *EGFR* and *KRAS* mutated/wild-type groups

**Figure 5:**
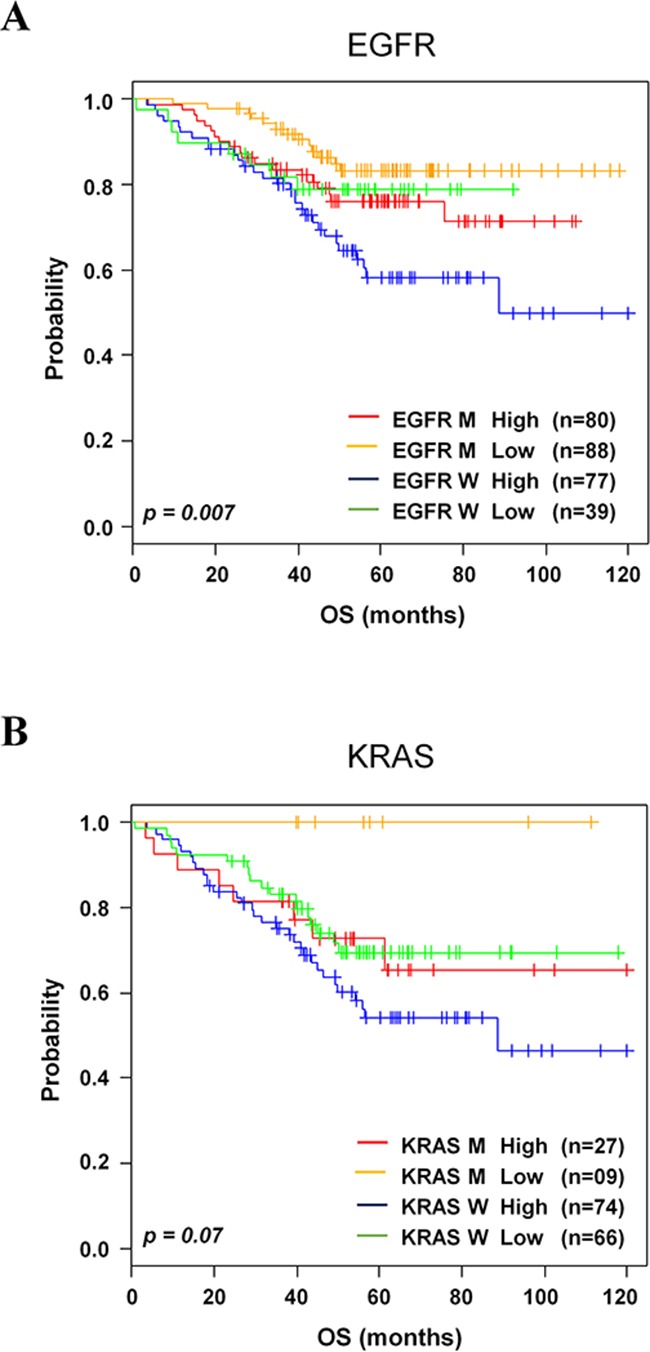
Kaplan-Meier survival analysis of the 8-gene signature with gene mutations **A**. Kaplan-Meier curves of patients in EGFR. **B**. KRAS in the validation data sets. Each group was classified by the 8-gene signature into low- and high-risk groups. The *p* values were computed by log-rank test.

Accumulation of *EGFR* and *KRAS* genetic alterations leads to the pathogenesis of lung cancer [12–15]. Based on the information of these genetic alterations available in validation data sets GSE31210 and GSE29013/E-MTAB-923, we investigated whether the 8-gene signature could further stratify lung cancer patients. In association analysis using χ^2^ tests, the 8-gene signature was significantly interrelated with *EGFR* status (*p=*0.007, Figure 5A) but barely with *KRAS* status (*p=*0.07, Figure 5B). These results strongly supported that the 8-gene signature would be helpful for prediction of prognosis particularly with *EGFR* alteration in NSCLC patients.

### Association of the 8-gene signature with histological subtypes

**Figure 6:**
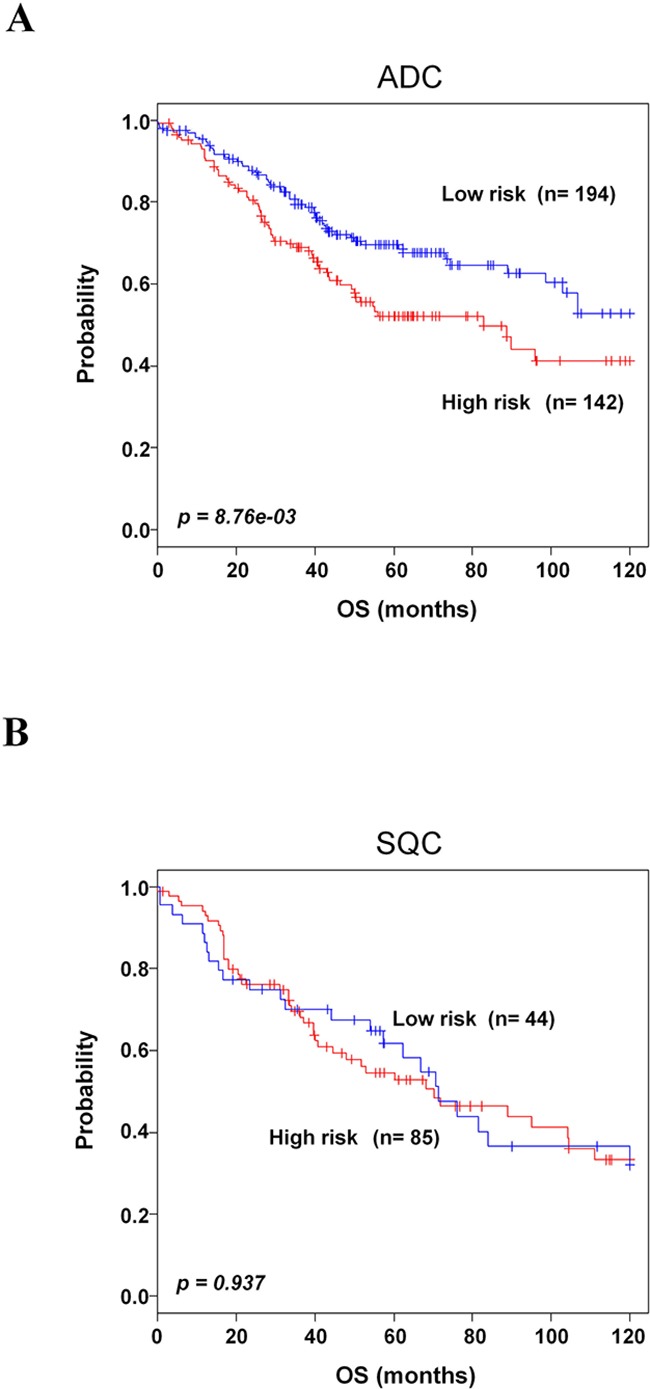
Kaplan-Meier survival analysis of the 8-gene signature with histological subtype **A**. Kaplan-Meier curves of patients with adenocarcinoma (ADC). **B**. Kaplan-Meier curves of patients with squamous cell carcinoma (SQC). Each group was classified by the 8-gene signature into low- and high-risk groups. The *p* values were computed by log-rank test.

To further determine whether lung cancer histology was associated with our 8-gene signature, we incorporated the gene signature into histological information in GSE30219, GSE29013, E-MTAB-923 and GSE42127. The 8-gene signature significantly classified the adenocarcinoma patients into low- and high-risk groups (*p=8.76e-03*, Figure 6A). However, it could not stratify the squamous cell carcinoma patients (Figure 6B).

## DISCUSSION

In this study, we have developed a novel 8-gene signature for NSCLC using computational approaches in tissues derived from patients. A supervised approach was integrated to construct the signature refined by LOOCV. Furthermore, the prognostic value of the 8-gene signature was determined in six microarray independent data sets (n=934) and one RNA-seq data set (n=543) patients. The robustness of the gene signature was supported by the high sensitivity (>0.90) and specificity (>0.90) values, and a significant association of predicted outcomes was found with patient prognosis in those data sets. Using univariate Cox analysis, the 8-gene signature was found to be one of the most reliable predictive factors for survival. Univariate and multivariate analyses performed after adjusting the clinical parameters showed a significant association of this prognostic gene signature with survival rate. Additionally, the 8-gene signature had the ability to identify stage II-III patients benefiting from ACT. Our gene signature strongly supported that 8 genes are also highly informative for prediction of patients with *EGFR*-mutated and wild type. These results suggest that our signature might be helpful in clinical management.

In clinical oncology, identification of individual patients who need ACT in NSCLC still represents a major concern. To date, only AJCC stage has been validated as the predictive factor to identify which patients should be treated with, or spared from chemotherapy. The benefit of ACT was previously demonstrated in patients at stage II and III [8, 9, 33]. In the context of survival benefit from ACT, a 15 gene-signature was first reported in resected NSCLC [24] in the JBR.10 trial [9]. Malignancy-risk gene signature was also developed as a predictive signature for ACT in lung cancer [34]. Recently, a 12-gene signature predicted ACT benefits with stage I-III NSCLC in two different data sets [35]. However, these gene signatures were studied in a small number of patients who received ACT and only tested on the JBR.10 trial data. None of the previously published findings showed a survival advantage in stage II-III patients. In our study, from the predictive point of view, the 8-gene signature has confirmed the potential to identify patients who would be likely to receive benefits from ACT. In subset analysis, the 8-gene signature clearly showed the benefit in stage II-III NSCLC patients. Patients in the high-risk group benefited significantly from ACT (HR, 1.47; 95% Cl, 1.01 to 2.14; *p=*0.044). In contrast, benefit from ACT was not statistically significant in low-risk group patients (HR, 0. 77; 95% Cl, 0.41 to 1.45; *p=*0.42). Our findings also confirmed the benefits of the ACT for patients with stage III. Therefore, we think that our 8-gene signature has the capability to facilitate clinical decisions for stage II-III NSCLC patients who might benefit from ACT.

Molecular alterations in *EGFR*, *KRAS*, and *ALK* genes are involved in lung cancer pathogenesis [12–15], but clinical use of these biomarkers is still a debatable issue. Because several prognostic gene signatures could not separate *EGFR*-mutated, *KRAS*-mutated, and wild-type patients into distinct subgroups, prognostic performance of these subgroups showed conflicting results [25, 36]. Consistent with previously published findings [17, 37], the 8-gene signature was interrelated with *EGFR* alteration. In contrast, the 8-gene signature was not able to be associated with *KRAS* alteration. At this time, we do not know why this happens, but we guess this discrepancy may be due to a small number of patients in this category [38]. We could not analyze our gene signature to classify patients with *ALK*-mutation due to small number of available data. Further studies are required to evaluate the 8-gene signature in response to chemotherapy in these mutation patients in independent and larger data sets. Therefore, our study demonstrated that the supervised analysis approach can identify patients with both mutation-specific and wild types into patients at higher risk with worse prognosis.

In the analyses by incorporating the 8-gene signature into histological information, our gene signature further stratified the patients with adenocarcinoma into high- and low-risk groups. However, unfortunately, the 8-gene signature could not significantly predict the prognosis for patients with squamous cell carcinoma. The 8-gene signature might imply the potential benefit of individual treatment in patients with adenocarcinoma, although we agree that it would not be enough to make a strong conclusion on the predictive power for squamous cell carcinoma due to the small number of patients.

Notably, some genes in the 8-gene signature (*STAT1*, *CLU*, *GTSE1* and *NUSAP1*) are involved in angiogenesis, invasion, migration, and proliferation. Overexpression of *STAT1* was observed in lung cancer progression [39]. *STAT1* promotes tumor growth by diverse processes that range from suppression of tumor immune surveillance and an increase in invasiveness/metastasis to acquisition of resistance against irradiation and chemotherapy [40]. It is also related to purinergic signaling which has immunologic consequences in patients with neoplastic disease [41]. *CLU* is upregulated after exposure to chemo- and radiotherapy in studies for lung cancer cell lines and animal models. In NSCLC prognostic research, *CLU*-positive patients with lung cancer had a better overall survival and disease-free survival than those with CLU-negative tumors [42]. *STAT1* and *CLU* are also involved in hypoxia and inflammation which are two inseparable hallmarks in tumorigenesis [43], indicating that they really play important roles in NSCLC pathogenesis. *GTSE1*, a negative regulator of p53, facilitates the proteasomal degradation of p53 during cellular recovery from DNA damage [44]. *NUSAP1* expression is positively correlated with tumor progression and recurrence [45, 46]. Thus, we read that these genes have significant roles in the NSCLC tumorigenesis. In our current study, their expression patterns in NSCLC patients of our current study were corresponded to results from the previous studies [39, 42, 47, 48]. In addition, our gene signature identified new promising biomarkers such as ATP binding cassette subfamily A member 8 (*ABCA8*), troponin T1 (*TNNT1*), ectonucleoside triphosphate diphosphohydrolase 3 (*ENTPD3*) and carboxypeptidase A3 (*CPA3*).

Here, we report the identification of the 8-gene signature by system biology approaches using highly reliable NSCLC data sets. The 8-gene signature predicted patients at high-risk of mortality in all validation data sets. Moreover, our gene signature predicted which patients would respond to ACT. In clinical context, the gene signature stratified patients into two distinct prognostic risk groups, and thus overcomes limitations in conventional classification. Therefore, the 8-gene signature can preferentially be valuable as an independent and accurate prognostic predictor and provides an opportunity for future clinical trial to test the benefit of chemotherapy in NSCLC patients.

## MATERIALS AND METHODS

### Patient and gene expression data

All data sets were downloaded from the National Center for Biotechnology Information Gene Expression Omnibus database (http://www.ncbi.nlm.nih.gov/geo) and Array express (https://www.ebi.ac.uk/arrayexpress/). Data were selected based on the chip type [Affymetrix U133 Plus 2.0 (GPL570), HG-133A (GPL96) and Illumina HumanWG-6 v3.0 expression beadchip (GPL6884)] (Supplementary Table S1). Raw data were preprocessed using robust multiarray averaging (RMA) method for normalization. GSE50081 (n=181, University Health Network) [29] was used as the training data set. GSE31210 (n=226, National Cancer Center Hospital) [25], GSE30219 (n=285, INSERM-UJF) [31], GSE29013 (n=55, UT Southwestern Medical Center) [32], E-MTAB-923 (n=90, French National League against Cancer) [36], GSE68465 (n=104, Memorial Sloan-Kettering Cancer Center) [23] and GSE42127 (n=174, UT Southwestern Medical Center) [35] were used as validation data sets (Supplementary Table S3). To test the prognostic significance of gene signature, only gene expression data with available survival data were used. ACT information was available for 170 patients from the validation data sets.

### Development of the prognostic gene expression signature

A gene expression signature to predict prognostic risk was developed from the training data set (GSE50081). Gene expression and overall survival (OS) data were combined to build a gene expression profiling-based survival classifier. The 54,675 probe sets were filtered by gene filtration using at least 2 absolute value of log2 scale, which represented the same gene expression level. The univariate Cox proportional hazard regression (p < 0.001) was then used to identify OS-associated gene expression signature from the training data set. Regarding prediction of prognosis, genes from the survival signature were applied to the survival risk prediction analysis [49]. This method used the principal component from the training data set and produced a prognostic index (PI) for each patient. The PI was computed by the formula ∑_i_w_i_ x_i_ - 0.00895 where w_i_ and x_i_ were the weight and logged gene expression for the i-th gene, respectively. Patients were classified into two groups based on a median prognostic index of 0.047018. Patients were assigned to the high-risk group if their prognostic indices were greater than the median value, whereas the low-risk group was composed of patients with prognostic indices that were equivalent to or less than the median value.

### Validation of the prognostic signature

The validation of the gene signature was accomplished on independent data sets. Gene expression data from different data sets were adjusted individually by subtracting the median expression value across the samples. To further refine this model and to sub-stratify the predicted outcomes, Compound Covariate Predictor (CCP) was utilized as a class prediction algorithm [50]. The robustness was estimated by the misclassification rate that was determined during the leave-one-out cross-validation (LOOCV) in the training data set.

Kaplan-Meier survival analyses were performed after patient classification into two risk groups, and Chi-square (χ^2^) and log-rank tests were used to evaluate the survival risk between two predicted subgroups of patients. The univariate and multivariate Cox proportional hazard regression analyses were used to evaluate independent prognostic factors associated with survival. Gene signature, stage, smoking, gender, and age were employed as covariates.

### Statistical methods of microarray data

Microarray data and heatmap were analyzed using BRB-Array Tools Version 3.0 (http://linus.nci.nih.gov/BRB-ArrayTools.html) [51]. All other statistical analyses were accomplished in the R language environment (http://www.r-project.org) and Statistical Package for Social Sciences (SPSS) software (version 20, SPSS Inc, Chicago, IL, USA). In all statistical analyses, *p* value of less than 0.05 was considered significant.

## SUPPLEMENTARY MATERIALS AND METHODS


