# Supplementary material for: An 8-gene signature for prediction of prognosis and chemoresponse in non-small cell lung cancer
Source: Oncotarget. 2016 Nov 15;7(52):86561–72. doi: 10.18632/oncotarget.13357 (PMC5349935; doi:10.18632/oncotarget.13357)
Supplement: Supplementary file 1 [file oncotarget-07-86561-s001.pdf]

An 8-gene signature for prediction of prognosis and chemoresponse in non-small cell lung cancer

SUPPLEMENTARY MATERIALS AND METHODS

Patient and gene expression data

Prognostic index (PI) for validation data sets was computed by the formula $\sum_i w_i x_i$ - 0.823 where w_i and x_i were the weight and logged gene expression for the i-th gene, respectively. Patients were classified into two groups based on a median prognostic index of 0.047018. Patients were assigned to the low- and high-risk groups based on PI. In addition, to validate the 8-gene signature, RNA-sequencing data from TCGA (n=543) were downloaded from Cancer Browser (https://genome-cancer.ucsc.edu) and used as validation data set.

Validation of the prognostic signature

Kaplan-Meier survival analyses were performed after patient classification into two risk groups and log-rank tests were used to evaluate the survival benefit from chemotherapy between two predicted subgroups with stage I or II patients.

SUPPLEMENTARY FIGURES AND TABLES

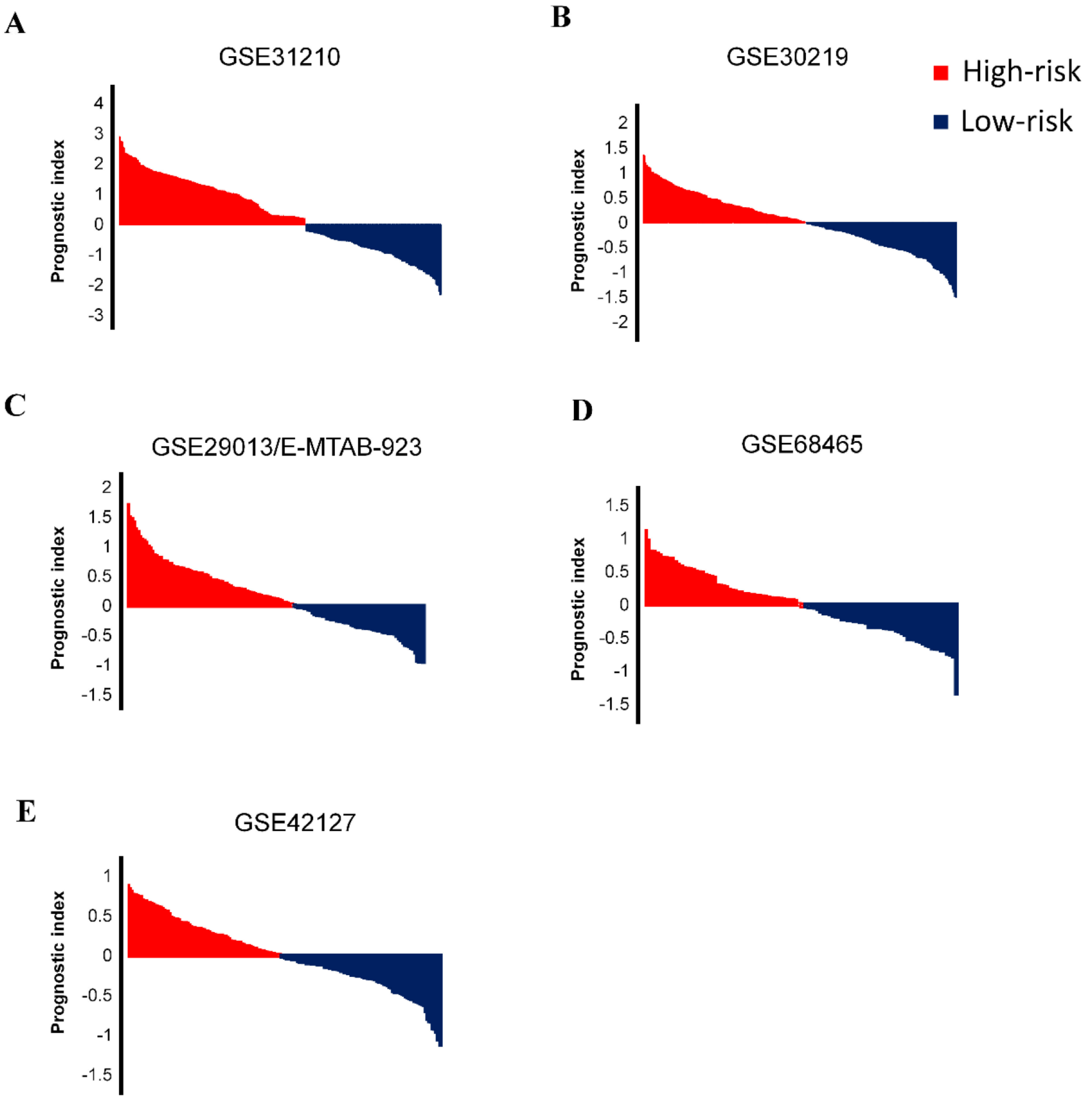

Supplementary Figure S1: Prognostic indices based on the 8-gene signature in the validation data sets. The relative prognostic indices were calculated according to the 8-gene signature expression of each patient in **A.** GSE31210, **B.** GSE30219, **C.** GSE29013/E-MTAB-923, **D.** GSE68465, and **E.** GSE42127.

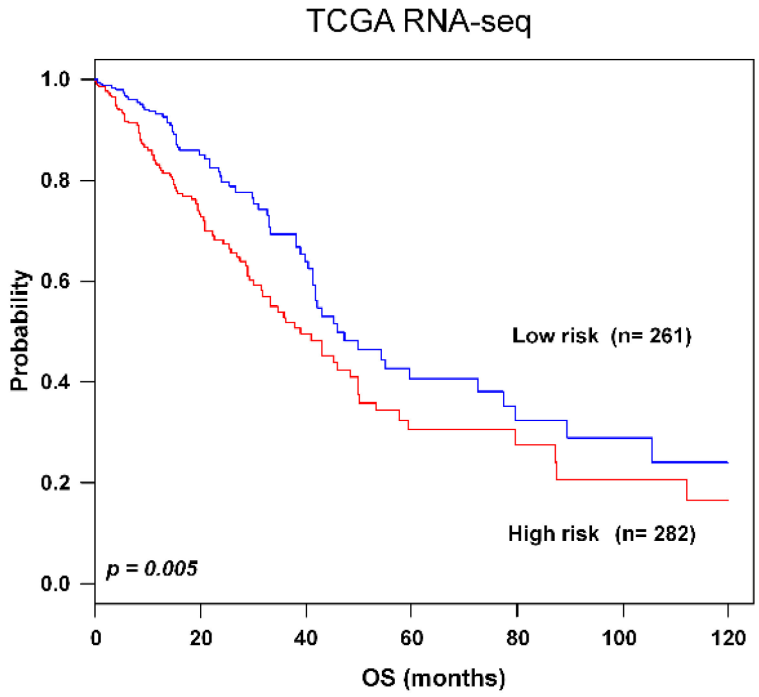

Supplementary Figure S2: Prognostic significance of the 8-gene signature in RNA-seq TCGA data set. Kaplan-Meier survival plots of overall survival (OS) of the two groups in the RNA-seq data. The *p* values were computed by the log-rank test.

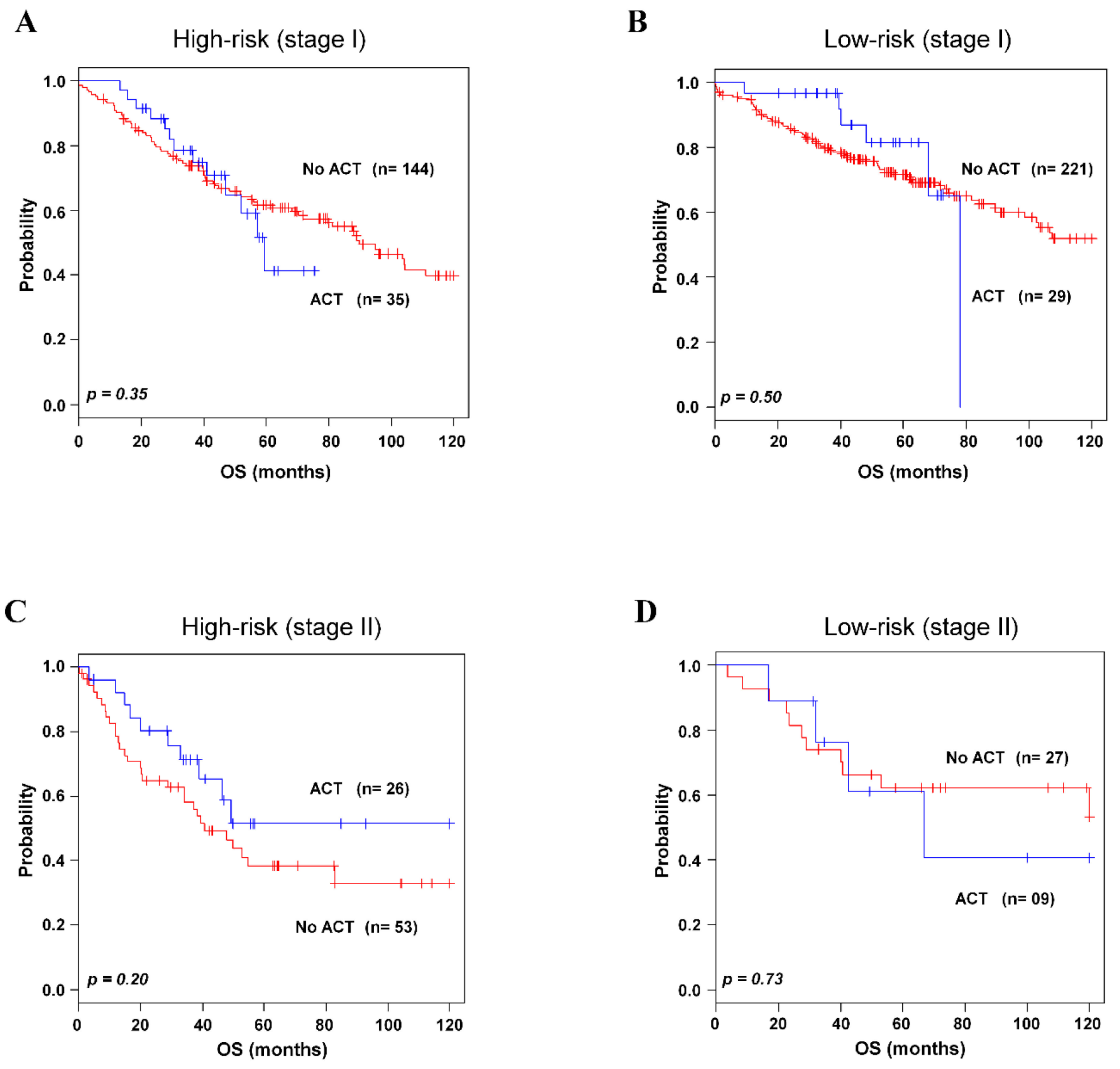

Supplementary Figure S3: Kaplan-Meier survival analysis of adjuvant chemotherapy. A-B. Kaplan-Meier plots of patients in high- and low-risk groups with stage I. **C-D.** Kaplan-Meier plots of patients in high- and low-risk groups with stage II. Patients were plotted according to presence and absence of adjuvant chemotherapy. The *p* values were computed by log-rank test.

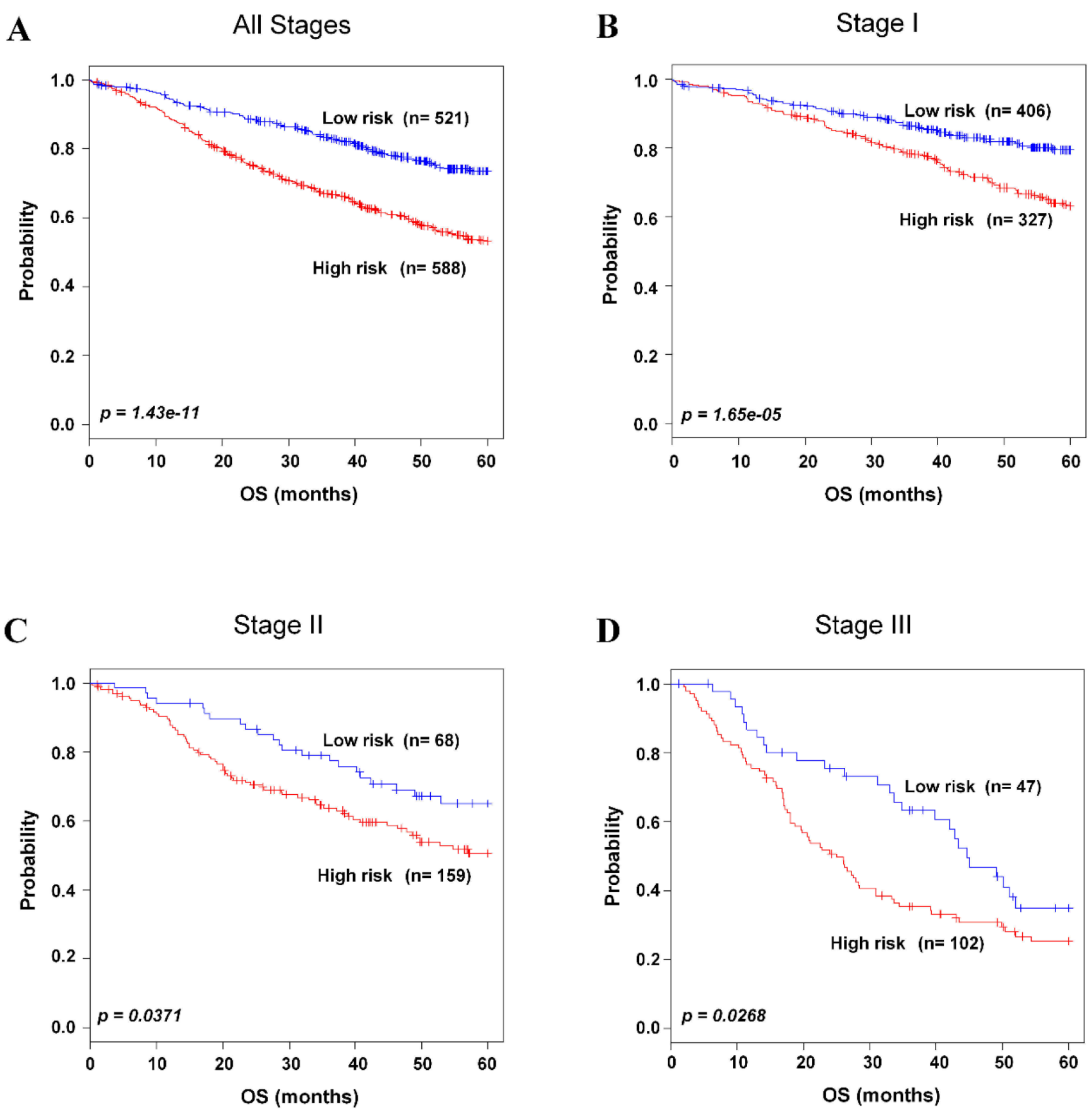

Supplementary Figure S4: Kaplan-Meier survival analysis of the 8-gene signature with stages in 5-year overall survival. A. Patients in all stages in the combined training and validation data sets. **B.** Patients in stage I in the combined training and validation data sets. **C.** Patients in stage II in the combined training and validation data sets. **D.** Patients in stage III in the validation data sets were classified by the 8-gene signature into low- and high-risk groups. The *p* values were computed by log-rank test.

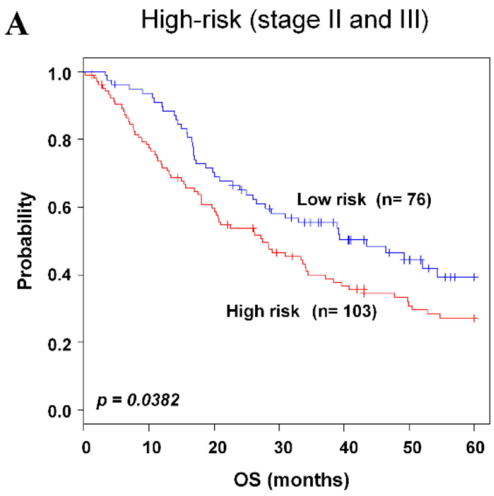

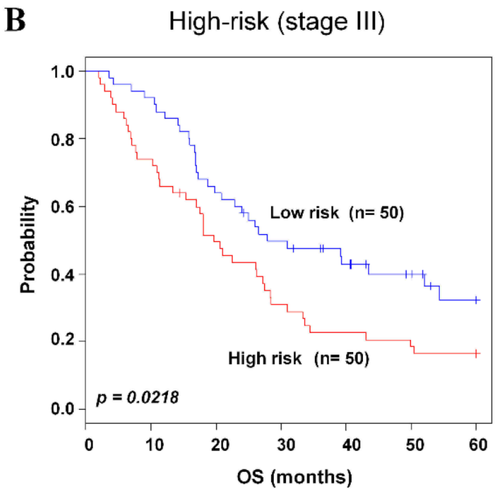

Supplementary Figure S5: Kaplan-Meier survival analysis of the 8-gene signature with adjuvant chemotherapy in 5-year overall survival. Patients from combined validation data sets with available adjuvant chemotherapy (ACT) data were included for analysis. **A-B.** Patients in high- and low-risk groups with chemotherapy in stage II-III. **C-D.** Patients in high- and low-risk groups with chemotherapy in stage III. Patients were plotted in the presence or absence of ACT. The *p* values were computed by log-rank test.

Supplementary Table S1: NSCLC microarray data sets

GEO Number	Origin/Year	Chip type	References
GSE50081	Canada, 2014	HG-U133 Plus_2	Der *et al*,
GSE31210	Japan, 2012	HG-U133_Plus_2	Okayama *et al*,
GSE30219	USA, 2013	HG-U133_Plus_2	Rousseaux *et al*,
GSE29013	USA, 2011	HG-U133_Plus_2	Xie *et al*,
E-MTAB-923	France, 2012	HG-U133_Plus_2	Fouret *et al*,
GSE68465	USA, 2008	HG-U133A	Shedden *et al,*
GSE42127	USA, 2013	IlluminaHuman-WG6 V3	Tang *et al,*

Supplementary Table S2: List of 8 genes in the prognostic expression signature

Symbol	Name	Entrez ID	Accession	Hazard Ratio	Low-risk	High-risk
ABCA8	ATP-binding cassette, sub-family A (ABC1), member 8	10351	NM_007168	0.713	Upregulated	Downregulated
CLU	Clusterin	1191	AI982754	0.703	Upregulated	Downregulated
CPA3	Carboxypeptidase A3 (mast cell)	1359	NM_001870	0.732	Upregulated	Downregulated
ENTPD3	Ectonucleoside triphosphate diphosphohydrolase 3	956	NM_001248	1.46	Upregulated	Downregulated
GTSE1	G-2 and S-phase expressed 1	51512	AI340239	0.876	Downregulated	Upregulated
NUSAP1	Nucleolar and spindle associated protein 1	51203	NM_018454	0.67	Downregulated	Upregulated
STAT1	Signal transducer and activator of transcription 1, 91kDa	6772	BC002704	1.541	Downregulated	Upregulated
TNNT1	Troponin T type 1 (skeletal, slow)	7138	AJ011712	1.301	Downregulated	Upregulated

Supplementary Table S3: Clinical characteristics of NSCLC patients in the training and validation data sets

	Training		Validation			
**Variables**	**GSE50081**	**GSE31210**	**GSE30219**	**GSE29013/E-MTAB-923**	**GSE68465**	**GSE42127**
No. of patients	181	226	285	145	104	174
Gender						
Male/Female	98/83	105/121	245/40	52/93	37/67	91/83
Median Age (years)	69.77	61	62	65	65	66.15
Disease stage						
I	127	168	185	78	63	112
II	54	58	40	23	20	32
III			54	44	21	30
NA			6			
Chemotherapy						
Yes			25	65	31	49
No			255	69	73	125
NA	181	226	5	11		
Smoking Status						
Ever smokers	136	111	255	92	83	
Never smokers	24	115	24	53	19	
NA	21		6		2	
Histological type						
ADC	128		85	120		131
SQC	43		61	25		43
Others	10		139			
No. of deaths	75	35	184	57	39	62

ADC: Adenocarcinoma, SQC: Squamous carcinoma, NA: Not Available

Supplementary Table S4: Clinicopathological characteristics of NSCLC patients in two risk groups of the training set

Variables		Total	Low-risk	High-risk	*p* (χ^2-test)
**Number of patients**		**181**	**89**	**92**	
Gender	Male	98	42	56	0.074
	Female	83	47	36	
Age	<60	24	13	11	0.664
	≥60	157	76	81	
Smoking	Ever	136	61	75	0.003
	Never	24	19	5	
AJCC stage	I	127	71	56	0.006
	II	54	18	36	
OS	0	106	66	40	4.02e-5
	1	75	23	52	

Supplementary Table S5: Univariate and multivariate Cox proportional hazard regression analyses in the validation data sets

Variable	Univariate			Multivariate		
	HR	95% CL	*p*-value	HR	95% CL	*p*-value
**Age**	1.41	1.13-1.76	0.002	1.30	1.03-1.69	0.039
**Gender**	0.60	0.48-0.74	6.15E-6	0.71	0.53-0.95	0.022
**Smoking (N vs E)[a]**	2.198	1.62-2.98	3.93E-7	1.26	0.83-1.91	0.273
**ACT**	1.20	0.93-1.55	0.155	0.91	0.67-1.25	0.584
**Stage (I, II, III)**	1.93	1.71-2.18	1.79e-26	1.75	1.50-2.05	2.18e-12
**8-gene signature**	1.77	1.43-2.20	1.71e-7	1.34	1.02-1.77	0.034

[a] N; Never smoking, E; Ever smoking
